# Coaching Intensity, Adherence to Essential Birth Practices, and Health Outcomes in the BetterBirth Trial in Uttar Pradesh, India

**DOI:** 10.9745/GHSP-D-19-00317

**Published:** 2020-03-30

**Authors:** Dale A. Barnhart, Donna Spiegelman, Corwin M. Zigler, Nabihah Kara, Megan Marx Delaney, Tapan Kalita, Pinki Maji, Lisa R. Hirschhorn, Katherine E. A. Semrau

**Affiliations:** a Harvard T.H. Chan School of Public Health, Boston, MA, USA.; b Center for Methods in Implementation and Prevention Science and Department of Biostatistics, Yale School of Public Health, New Haven, CT, USA.; c University of Texas, Austin, TX, USA.; d Dell Medical School, Austin, TX, USA.; e Ariadne Labs, Boston, MA, USA.; f Population Services International, Lucknow, Uttar Pradesh, India.; g Access Health International, Hyderabad, Telangana, India.; h Northwestern University Feinberg School of Medicine, Chicago, IL, USA.; i Brigham and Women's Hospital, Boston, MA, USA.; j Harvard Medical School, Boston, MA, USA.

## Abstract

**Background:**

Coaching can improve the quality of care in primary-level birth facilities and promote birth attendant adherence to essential birth practices (EBPs) that reduce maternal and perinatal mortality. The intensity of coaching needed to promote and sustain behavior change is unknown. We investigated the relationship between coaching intensity, EBP adherence, and maternal and perinatal health outcomes using data from the BetterBirth Trial, which assessed the impact of a complex, coaching-based implementation of the World Health Organization's Safe Childbirth Checklist in Uttar Pradesh, India.

**Methods:**

For each birth, we defined multiple coaching intensity metrics, including coaching frequency (coaching visits per month), cumulative coaching (total coaching visits accrued during the intervention), and scheduling adherence (coaching delivered as scheduled). We considered coaching delivered at both facility and birth attendant levels. We assessed the association between coaching intensity and birth attendant adherence to 18 EBPs and with maternal and perinatal health outcomes using regression models.

**Results:**

Coaching frequency was associated with modestly increased EBP adherence. Delivering 6 coaching visits per month to facilities was associated with adherence to 1.3 additional EBPs (95% confidence interval [CI]=0.6, 1.9). High-frequency coaching delivered with high coverage among birth attendants was associated with greater improvements: providing 70% of birth attendants at a facility with at least 1 visit per month was associated with adherence to 2.0 additional EBPs (95% CI=1.0, 2.9). Neither cumulative coaching nor scheduling adherence was associated with EBP adherence. Coaching was generally not associated with health outcomes, possibly due to the small magnitude of association between coaching and EBP adherence.

**Conclusions:**

Frequent coaching may promote behavior change, especially if delivered with high coverage among birth attendants. However, the effects of coaching were modest and did not persist over time, suggesting that future coaching-based interventions should explore providing frequent coaching for longer periods.

## Abstract (precis)

Frequent coaching was associated with increased adherence to evidence-based essential birth practices among birth attendants but not with improved maternal and perinatal health outcomes in the BetterBirth Trial, which assessed the impact of a complex intervention to implement the World Health Organization's Safe Childbirth Checklist. To promote sustainable behavior change, future coaching-based interventions may need to explore cost-effective, feasible mechanisms for providing more frequent coaching delivered with high coverage among health care workers for longer durations.

## INTRODUCTION

Rates of maternal and neonatal mortality in low- and middle-income countries can be more than 10 times higher than in high-income countries.^1^^,^^2^ Despite global increases in facility-based deliveries, progress in reducing the rates of these preventable deaths has been slower than expected due to poor quality of care in health facilities and poor adherence to evidence-based practices among birth attendants.^3^^–^^7^ Improving the quality of care at birth facilities has the potential to avert 531,000 stillbirths, 1.3 million newborn deaths, and 112,000 maternal deaths each year.^8^ However, evidence-based strategies for improving the quality of care in birth facilities are lacking. Providing training alone can increase knowledge of evidence-based practices but does not necessarily translate into meaningful improvements in quality of care.^9^^,^^10^ Consequently, additional strategies are needed to improve the quality of intrapartum and postpartum care.

Coaching is one strategy to promote birth attendant behavior change. The coaching process helps individuals use their existing skills, resources, and training to improve their performance and achieve personalized goals.^11^^,^^12^ Typically, coaching focuses on individual behavior change, but it can also be directed toward addressing systemic problems. Unlike traditional supervision, which is a hierarchical process where a leader is accountable for the activities of a group or individual,^13^ or mentoring, which is focused more broadly on professional and personal development, coaching is individual-focused, task-oriented, and performance-driven.^14^ To improve performance, coaches use multiple approaches, including modeling desired behaviors, providing supportive supervision, providing auditing and feedback, and promoting problem solving.^15^ These strategies are effective at improving quality of care in low- and middle-income countries across a variety of clinical areas,^16^^,^^17^ including Integrated Management of Childhood Illness,^18^^,^^19^ drug management and prescription practices,^20^^,^^21^ primary care,^22^ malaria case management,^23^ voluntary male circumcision,^24^ and reproductive health.^25^^,^^26^

Although some studies have reported associations between increased intensity of coaching-related activities and improved quality of care,^21^^–^^23^ the optimal coaching intensity needed to promote and sustain behavior change is unknown. Coaching intensity can be quantified across multiple domains, including frequency (e.g., 2 coaching visits per week); duration (e.g., 6 weeks of coaching); and cumulative dose, which reflects both the frequency and the duration of the intervention (e.g., 2 sessions per week for 6 weeks equals 12 cumulative visits).^27^ Once the desired coaching regimen has been determined, coaching fidelity can also be described in terms of scheduling adherence or the extent to which the coaching regimen is delivered in accordance with the intended schedule.^28^ Additional dimensions of coaching intensity exist (Box), and fidelity could also be described in terms of these other domains. Understanding which domains of coaching intensity are most strongly associated with quality of care improvements can identify coaching regimens that are optimized to promote behavior change and, ultimately, to improve health outcomes.

BOX **Coaching Intensity Domains**

**Coaching Form**^a^: coaching delivery method, including the coach's identity and experience level (e.g., peer coaching, expert coaching) and the strategies the coach used to generate behavior change (e.g., role playing, motivational support)

**Coaching Quality**^a^: coach's ability to correctly and consistently use coaching strategies to generate behavior change

**Coaching Frequency**: number of coaching sessions delivered over a specific duration of time (e.g., 2 coaching visits per week)

**Coaching Duration**^a^: time period during which coaching is delivered (e.g., 6 weeks of coaching)

**Cumulative Coaching**: accrual of exposure to coaching over time that is determined by both coaching frequency and coaching duration (e.g., 2 sessions per week for 6 weeks equals 12 cumulative visits)

^a^Coaching intensity domain not covered in this analysis.

One coaching-based intervention designed to improve the quality of care provided to mothers and newborns during facility-based childbirth is the BetterBirth Program. Although this intervention did not reduce maternal morbidity or maternal and perinatal mortality in a recent matched-pair, cluster-randomized trial conducted in Uttar Pradesh, India, it increased birth attendant adherence to 18 essential birth practices (EBPs) believed by experts to prevent or successfully manage complications during facility-based deliveries from an average of 7.9 in the control arm to an average of 11.1 in the intervention arm.^29^

In this article, we used data from the intervention arm of the BetterBirth Trial to assess the relationship between coaching frequency, cumulative coaching, and scheduling adherence with birth attendant adherence to EBPs and maternal and perinatal health outcomes. By investigating multiple dimensions of coaching intensity, we aimed to provide insights into the optimal coaching regimen for future coaching-based interventions.

By investigating dimensions of coaching intensity, we aimed to provide insights into the optimal coaching regimen for future interventions.

## METHODS

### Intervention

The BetterBirth Program was designed to promote the use of the World Health Organization's Safe Childbirth Checklist (SCC), a 28-item tool intended to assist birth attendants in performing EBPs. To ensure EBPs are completed in time to avoid potential complications, these items are organized into 4 “pause points”: (1) on admission, (2) just before pushing, (3) within 1 hour after birth, and (4) before discharge. When using the checklist, birth attendants can either first read the item and then complete the task or first provide care and then review the checklist to confirm that all tasks for that pause point have been completed.^30^ Coaching was recommended as a core component of SCC implementation packages since the checklist's initial development^31^ and was a major feature of the BetterBirth Program's multicomponent implementation package.

The BetterBirth Program used an engage-launch-support model, which has been previously described in detail.^32^^–^^34^ Briefly, district- and facility-level leadership were introduced to the BetterBirth program and engaged to identify priority areas for improvement related to the SCC. Each facility held an educational and motivational launch event to train birth attendants on using the SCC. Finally, ongoing coaching and data feedback were used to support behavior change.

Birth attendants, who were primarily auxiliary nurse midwives or general nurses in the labor and delivery wards, received peer-to-peer coaching from study staff who were nurses with training in childbirth and at least 2 years of experience in delivery. These coaching relationships were designed to be collaborative and were not designed to replace the existing traditional supervision structure. In practice, coaches' training, age, and years of experience were similar to that of the nurse birth attendants, who comprised approximately 78% of the birth attendant population. However, about 16% of the birth attendant population were auxiliary nurse midwives, who were older and had fewer years of formal training but more years of experience, and approximately 7% were lady medical officers, who were trained as physicians.^35^

Coaches' clinical skills were assessed via interviews during the recruitment process. Coaches received 5–7 days of skills-based training, which emphasized coaching skills, including relationship building; verbal and nonverbal interpersonal communication; handling difficult persons or situations; observation, listening, and speaking skills; and prioritizing and setting goals with frontline health workers. The training program for coaches also included a physician-led review of government guidelines around skilled birth attendants.

Coaching followed an “opportunity-ability-motivations-supplies” framework adapted from previous behavior change models.^33^^,^^36^^,^^37^ In this framework, coaches motivated birth attendant behavior change using techniques such as storytelling and positive acknowledgment to emphasize the importance of adopting EBPs to meet national guidelines and save lives. They also observed deliveries, collected data, and provided real-time feedback on current adherence to EBPs; identified existing opportunity-, ability-, motivation-, or supply-related barriers to EBP adherence; and engaged in group problem solving to address these barriers. For example, if birth attendants were unable to complete EBPs because supplies or medications were missing, coaches would classify the issue as a supply-related barrier and advocate with administrators or pharmacists to obtain the supply. Other examples of how coaches addressed specific barriers can be found elsewhere.^33^^,^^38^ As part of the study design, coaches did not directly provide additional technical training or coach on clinical quality but did advocate with facility leaders (lady medical officers or medical officers in-charge) for additional training opportunities or engage in role playing to address ability-related barriers.

Birth attendant coaching was scheduled to occur twice per week during the first through fourth months of the intervention, once per week during the fifth and sixth months of the intervention, once every 2 weeks during the seventh month, and once per month in the eighth month for a total of 43 visits per health facility. Coaching visits were intended to last for the entire duration of a daytime shift (8 am to 5 pm). Additional details on the content of coaching sessions, perceptions around the credibility of the peer-coaching program, and the quality of the relationships between coaches and birth attendants have been published elsewhere.^34^ The same coaching schedule was planned for all facilities, regardless of delivery load or birth attendant staff size.

A parallel peer-to-peer coaching also occurred at the facility leadership level with facility leadership being coached by other physicians or public health professionals to help improve their abilities to provide leadership and supervision. In this manner, coaching was intended to support rather than substitute for existing supervision structures. Facility leadership coaching followed a similar, but less intensive, schedule for a total of 23 visits. To promote long-term sustainability, each site also designated a childbirth quality coordinator who was a well-respected facility-based staff member but not necessarily a supervisor or manager and was intended to serve as a long-term, facility-based coach and project champion. In practice, this role was often filled by a head nurse, medical officer in-charge, or pharmacist. This champion was intended to continue the practice of peer-coaching and not replace existing supervision practices.

### Trial Design and Study Setting

This implementation package was evaluated in a matched-pair, cluster-randomized trial that enrolled 120 primary-level health facilities in Uttar Pradesh, India, a region with high maternal (258/100,000 live births) and neonatal (49/1,000 live births) mortality.^39^

All facilities enrolled in the BetterBirth Trial were required to conduct at least 1,000 deliveries per year, have at least 3 birth attendants trained at the level of auxiliary nurse midwife or higher, have no concurrent quality improvement or research programs, and have district and facility leadership who were willing to participate. Forty-six facilities were primary health centers, 56 were community health centers, and 18 were first referral units.^29^ Primary-level facilities should have had the capacity to provide basic emergency obstetric and newborn care but often lacked the necessary resources to do so.^40^ District hospitals were not included in this study. Eligible facilities were matched on baseline characteristics and randomized within pairs to receive either the coaching-based intervention or the current standard of care. Roll-out of the intervention was staggered across 5 geographically-defined research hubs centered in the urban areas of Agra, Gorakhpur, Lucknow, Meerut, and Varanasi. Full details on study procedures, including sample size calculations, can be found elsewhere.^41^

### Data Collection

At each facility, registers were used to document the admission date for each woman in labor and any instances of facility-based mortality and morbidity. Data on 7-day health outcomes were obtained using a call center, which contacted mothers and their families between 8 and 42 days postpartum, followed by home visits if neither the woman nor a family member was reached by phone after 22 days postpartum.^42^

In a convenience sample of births occurring in 30 facilities (15 intervention, 15 control) located in the Lucknow hub, which is in central Uttar Pradesh, additional direct observations of deliveries were conducted to collect data on birth attendant EBP adherence. Trained independent nurses observed and recorded EBP adherence using standardized data collection tools. Visits from independent data collectors occurred in addition to the coaching visits, which occurred in all intervention facilities. Unlike coaches, who used the opportunity-ability-motivation-supplies framework to improve birth attendant performance, study nurses who served as independent observers did not serve as coaches and did not intervene in clinical care. Data collection on EBP adherence occurred during 3 of the 4 pause points: on admission to facility, just before pushing, and within 1 hour after birth. However, practical considerations related to the timing and duration of labor prevented all births from being continuously observed from admission through discharge. Consequently, not all EBPs were observed for each birth. For intervention facilities, nurse coaches recorded the date of each coaching visit as well as the unique ID code for each birth attendant who was coached during that visit.

### Outcomes

We considered 2 types of outcomes: birth attendant EBP adherence and maternal and perinatal health outcomes. EBP adherence was measured as the number of EBPs that a birth attendant successfully completed of the 18 practices that the World Health Organization recommends as essential for all mothers and newborns (Table 1).^29^^,^^30^ Previous research has suggested that this EBPs adherence metric is associated with reduced risk of perinatal mortality in this setting.^43^

**TABLE 1.** Eighteen Essential Birth Practices From the World Health Organization Safe Childbirth Checklist^a^

At Admission	Before Pushing	After Birth	Any Time
Partograph started	Hand hygiene	Oxytocin administered within 1 minute	Maternal temperature taken
Birth companion present	Clean towel available	Birth companion present	Maternal blood pressure taken
	Clean blade available	Baby weighed	
	Cord tie available	Baby temperature taken	
	Mucus extractor available	Skin-to-skin warming initiated	
	Neonatal bag available	Skin-to-skin warming maintained for 1 hour	
	Clean pads available	Breastfeeding initiated	

a Independent observers assessed the birth attendant's adherence to essential birth practices but not their technical skill or quality in performing the practice.

Mother-baby dyads were included as a birth in our EBP analysis if they occurred at 1 of the 15 intervention facilities where EBP adherence data were collected, occurred after the start of coaching at that facility, and were directly observed during admission to facility, just before pushing, and within 1 hour after birth such that adherence to all 18 practices was recorded. Our analysis was conducted exclusively among intervention sites to focus on likely effects of birth attendant coaching without potential confounding from other components of the complex intervention.

As in the main trial, our primary health outcome was a composite outcome of events occurring within 7 days after delivery that included severe maternal morbidity, defined as self-reported complications including seizures, loss of consciousness for more than 1 hour, fever with foul-smelling vaginal discharge, hemorrhage, or stroke; maternal mortality; or perinatal mortality, defined as stillbirth or death within the first 7 days of life. A secondary composite health outcome consisting of only 7-day maternal or perinatal mortality was also considered.^29^

Mother-baby dyads were included as a birth in the health outcomes analysis if they occurred in an intervention facility after the start of coaching, if mothers consented to follow-up, and if data on 7-day outcomes were obtained. As in the main trial, dyads were included in the 7-day outcome analysis even if they were transferred to a higher-level facility before delivery. Because the timing of direct observations of birth attendant adherence to EBPs (that occurred 0–8 and 13–17 months after the start of coaching) differed somewhat from the timing of call center activities (that continued from 0–13 months after the start of coaching), the EBP adherence sample is not a subset of the health outcomes sample. However, some births appear in both samples.

### Coaching Intensity

For each birth, we calculated metrics that reflected multiple domains of coaching intensity, including coaching frequency, cumulative coaching, and scheduling adherence. These metrics were based on the dates of the peer-to-peer birth attendant coaching visits that had occurred at a given facility before each birth. For coaching frequency, we assigned each birth a coaching intensity equal to the number of coaching visits occurring at that facility in the 30 days before the admission date (visits in the past month).

Because we hypothesized that the impact of coaching on birth-related outcomes would be stronger when we considered the intensity of coaching provided to the birth attendants who conducted the deliveries rather than to the facility as a whole, we also created coaching frequency metrics that reflected coaching delivered at the birth attendant level. In this study, it was not possible to identify which birth attendant conducted a specific delivery, so we created coaching metrics that reflected the delivery of coaching among all birth attendants working at a single facility. These metrics included the average number of visits in the past month among birth attendants, the percentage of birth attendants receiving at least 1 visit in the past month, and the standard deviation of coaching visits in the past month among birth attendants. We hypothesized that facilities would experience more benefits from coaching if birth attendants had, on average, a greater number of visits in the past month, higher coaching coverage (percentage of birth attendants receiving at least 1 visit in past month), and a more equal distribution of coaching visits among birth attendants (lower standard deviation in visits among birth attendants in the past month).

All metrics reflecting coaching delivered at the birth attendant level were calculated under the assumption that the birth attendants listed in the coaching database reflected a complete list of birth attendants employed by the facility over the course of the intervention. These metrics did not consider staff turnover, which was assumed to be minimal over the intervention period. We also explored coaching frequency metrics calculated over a 1-week, rather than a 1-month, time horizon. However, since these 2 time windows produced similar results, we have presented only the results for the 1-month time horizon. Results for the 1-week time horizon can be found in the Supplemental Tables.

For cumulative coaching, we assigned each birth a coaching intensity equal to the total number of coaching visits accrued at the facility between the start of program and the admission date (total visits). As with coaching frequency, we believed that (a) coaching delivered at the birth attendant level would have a greater impact on birth attendant behavior change and health outcomes than coaching delivered at the facility level and (b) facilities with higher coverage of coaching at the birth attendant level would experience greater benefits from coaching. Therefore, for each birth we also calculated the mean number of visits accrued among birth attendants between the start of the program and the admission date and the standard deviation of coaching visits among birth attendants.

Scheduling adherence was defined according to the prescribed frontline coaching schedule of attaining at least 2 visits per week during the first 4 months of the intervention, at least 1 visit per week during the fifth and sixth months, at least 1 visit every 2 weeks during the seventh month, and at least 1 visit per month during the eighth month. Current scheduling nonadherence was a binary variable reflecting whether the date of admission occurred on a day when the facility had deviated from this schedule. Cumulative scheduling nonadherence reflected the total number of nonadherent days accrued between the start of program and the date of admission. For example, if a facility had been 3 days late for its first coaching visit and 4 days late for its second coaching visit, then subsequent births would receive a cumulative scheduling nonadherence value of 7.

For each birth, we calculated the mean number of visits accrued among birth attendants between the start of the program and the admission date and the standard deviation of coaching visits among birth attendants.

### Statistical Methods

Because the BetterBirth Program prescribed high-frequency coaching early in the intervention and gradually reduced the frequency of coaching over time, there were strong correlations among coaching frequency metrics, cumulative coaching metrics, and time since the start of the intervention. We reported the mean and standard deviation for each coaching metric and explored correlations between coaching metrics graphically and using Spearman correlation coefficients. To assess associations between each metric of coaching intensity and the outcomes of interest, we used generalized linear models and accounted for clustering at the facility level by estimating standard errors using the empirical variance with an exchangeable working covariance structure.^44^

For EBP adherence, we estimated the change in the number of EBPs that birth attendants adhered to associated with each coaching intensity metric using an identity link and a normal distribution. For binary health outcomes, we estimated the risk ratios associated with each coaching intensity metric using a log link and a binomial distribution.^45^ Because coaching metrics had very different ranges (e.g., total coaching ranged from 1 to 47 and percentage of birth attendants receiving at least 10 visits ranged from 0% to 100%), we reported effect sizes associated with increasing each of the coaching metrics from their 25th percentile to their 75th percentile, or by 1 interquartile range. These percentiles were calculated in the health outcomes dataset. Where relevant, we also reported effect sizes for a 1-unit increase.

For all models, we used robust score tests to assess the statistical significance of model parameters.^46^ Our primary models adjusted for facility-level covariates, including research hub location; being located in a high-priority district, a designation used by the Indian government to identify districts with a high overall burden of mortality; distance to district hospital in kilometers; and number of skilled birth attendants at that facility. At the birth level, models also adjusted for whether or not the birth occurred on the same day as a coaching visit. We fit models for each coaching metric separately and also used stepwise regression to assess whether multiple coaching metrics should be included in the same model based on an α ≤.05 criterion for model entry and exit.

Because the effects of behavior change interventions often fade over time,^47^ a phenomenon that could render time since start of the intervention to act as a confounder that biases results against cumulative coaching metrics and in favor of coaching frequency metrics, in a secondary set of models we additionally adjusted for months since the start of the intervention. We tested for potential nonlinear relationships between months since the start of the intervention and our outcomes of interest using restricted cubic splines^48^ selected using a publicly available SAS macro.^49^

Finally, we assessed whether the association between coaching intensity metrics and EBP adherence or health outcomes changed over the course of the intervention by adding an interaction between each coaching metric and months since the start of the intervention to our models. Because of the strong collinearities between coaching metrics and months since the start of the intervention, several models produced statistically significant interaction terms that were not interpretable. To ensure interpretability, we reported results for these interaction models only if both the time-by-coaching interaction term and the overall effect of coaching based on the joint null hypothesis that both the main effect of coaching and its interaction term were zero, were statistically significant at the α=.05 level.

## RESULTS

### Study Population

**FIGURE 1:**
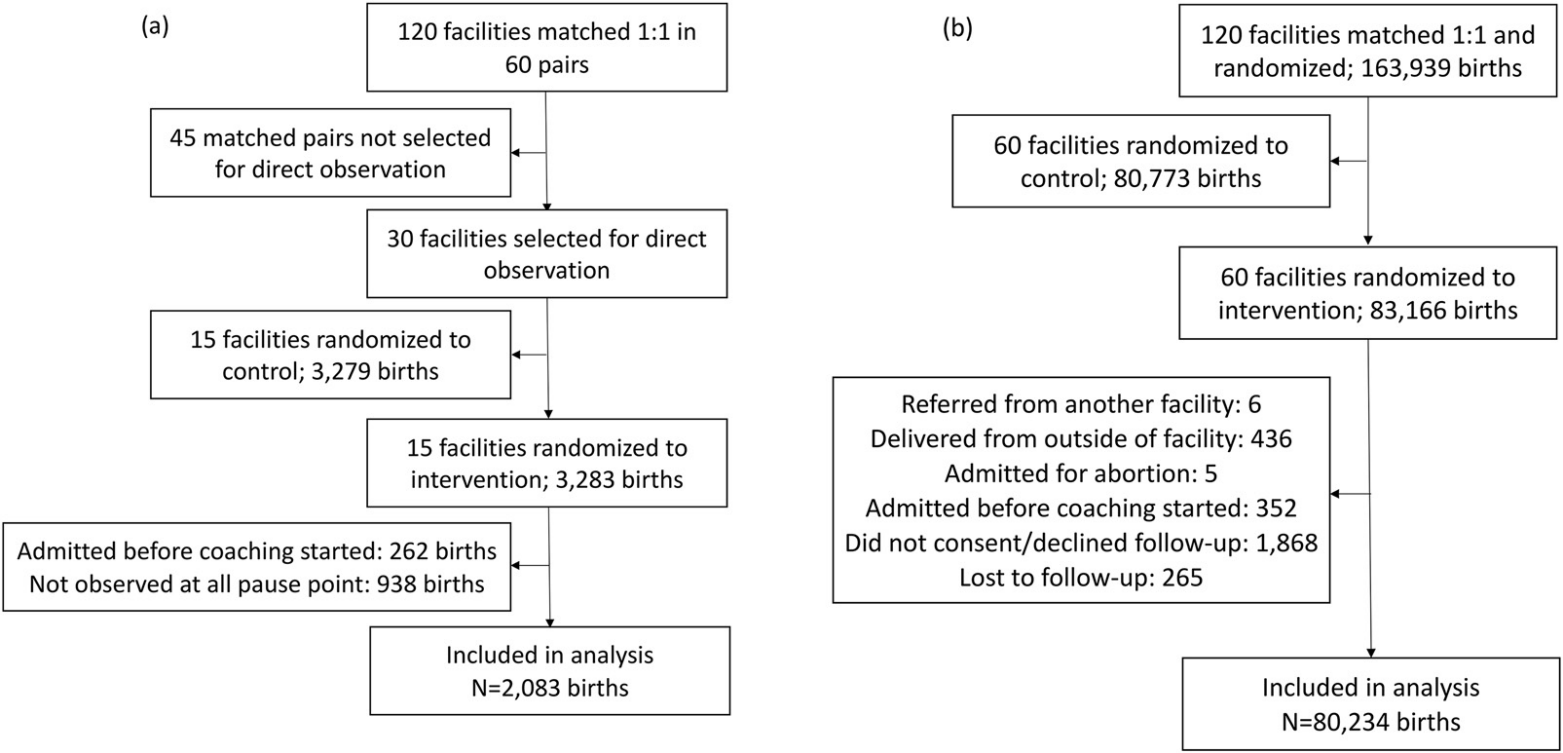
Study Populations from the BetterBirth Trial for Analysis on (a) Essential Birth Practice Adherence and (b) Health Outcomes,^a^ Uttar Pradesh, India ^a^Sample includes 436 births that were excluded from main the randomized controlled trial analysis due to being involved in baseline collection.

Data on EBP adherence at intervention facilities were collected for 3,283 births. We excluded 262 births that occurred before the start of the coaching intervention and 938 that were not observed for all 3 pause points for a final sample of 2,083 births (Figure 1a). Health outcomes data at intervention facilities were collected among 83,166 births. We excluded 6 deliveries referred from another facility, 436 deliveries that occurred after a study facility's obstetric services moved to a new location, 5 women admitted for abortion, 352 births that occurred before the start of coaching, 1,868 births for which patients did not consent to follow-up, and 265 births that were lost to follow-up for a final sample of 80,234 births (Figure 1b). An additional 457 births lacked complete data on maternal morbidities and were excluded from analyses of the primary composite outcome. The EBP adherence and health outcome samples overlapped by 1,100 births and shared many similarities (Table 2). However, facilities in the EBP adherence sample came exclusively from the Lucknow hub, located in the center of the state, and were more likely to be in a high-priority district. Due to differences in the timing of data collection in the 2 samples, births in the EBP adherence sample were less likely to have occurred on a coaching day.

**TABLE 2.** Descriptive Statistics for the EBP Adherence and Health Outcomes Study Populations

	EBP Adherence Sample	Health Outcomes Sample
**Facility-level variables**	**N=15**	**N=60**
Research hub, No. (%)		
Agra	–	9 (15.0)
Gorakhpur	–	11 (18.3)
Lucknow	15 (100.0)	19 (31.7)
Meerut	–	7 (11.7)
Varanasi	–	14 (23.3)
High priority district, No. (%)	7 (46.7)	7 (11.7)
Distance to district hospital (km), mean (SD)	29.5 (12.0)	29.5 (14.0)
Number of skilled birth attendants, mean (SD)	4.5 (1.1)	4.4 (1.2)
Annual delivery load, mean (SD)	1,795 (468.0)	1,599 (435.0)
**Birth-level variables**	**N=2,083**	**N=80,234**
Birth occurred on coaching day, No. (%)	107 (5.1)	7,533 (9.4)
Months since intervention started at facility, mean (SD)	8.5 (5.8)	6.7 (2.8)
EBP adherence (of 18 practices), mean (SD)	12.1 (2.4)	–
Primary composite,^a^ No. (%)	–	12,062 (15.0)
Secondary composite, No. (%)	–	3,907 (4.9)

Abbreviations: EBP, essential birth practices; SD, standard deviation.

a 457 births are missing data on maternal morbidity, and therefore, are missing data on the primary composite outcome.

### Coaching Intensity

**FIGURE 2:**
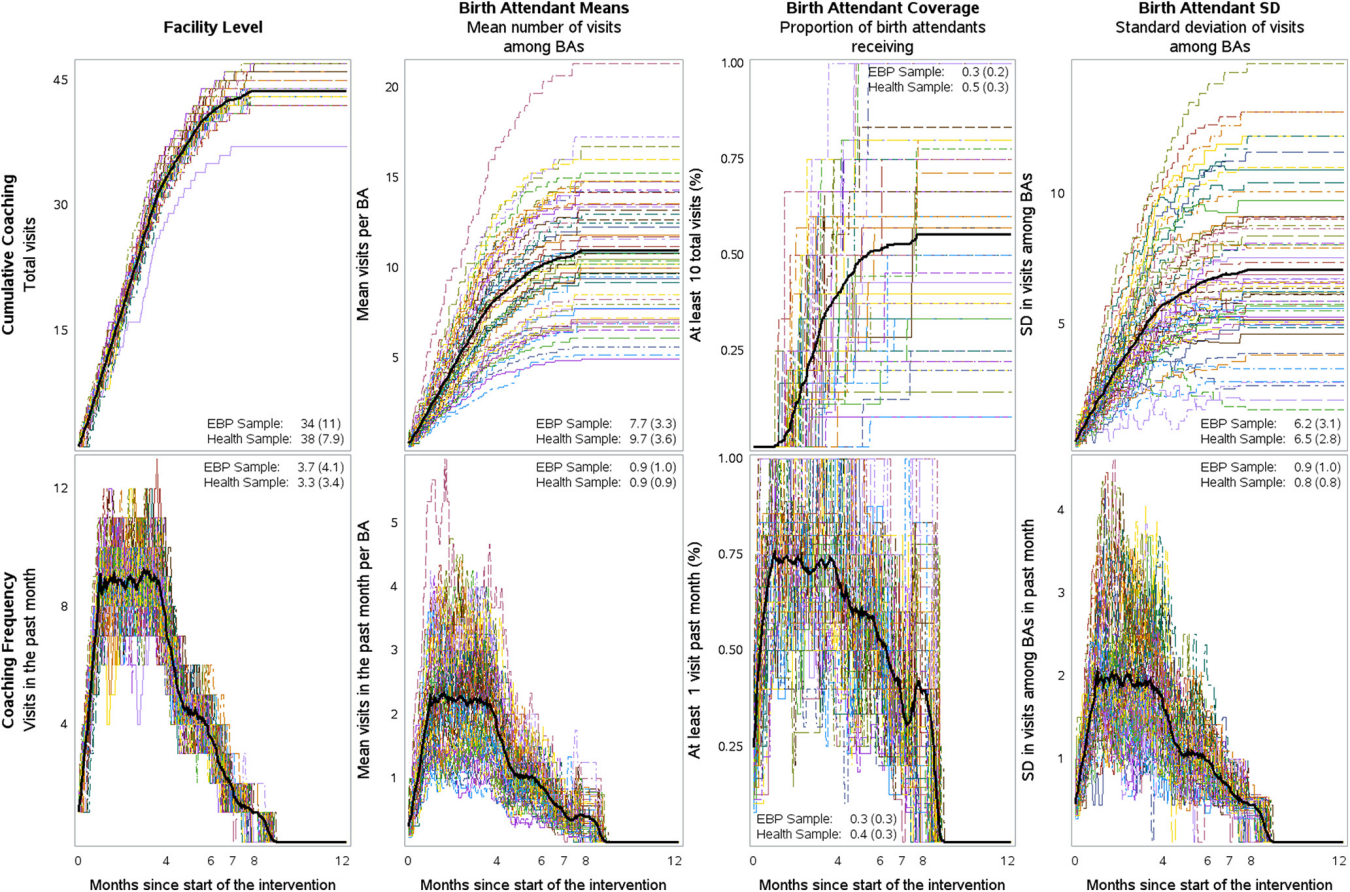
Coaching Intensity Over Time^a^ ^a^Each colored line reflects the coaching intensity at a given facility over time with the bolded black line reflecting the average coaching intensity across all facilities. Each panel provides the mean and (standard deviation) for the exposure in the EBP adherence and health outcome samples. Abbreviations: BA, birth attendant; SD, standard deviation.

Fidelity to the coaching schedule was very high. By the end of the intervention, of the 60 facilities, 53 (88%) of facilities reached the target of 43 total coaching visits, 6 (10%) reached 42 visits, and 1 (2%) facility reached 37 visits. However, fidelity at the facility level did not necessarily translate into delivery of high-coverage coaching among birth attendants. Although birth attendants received, on average, 10 coaching visits by the end of the intervention, 34% received fewer than 5 visits. Figure 2 shows the changes in coaching metrics over time as well as the global mean and standard deviation for each coaching metric. As would be expected based on the prescribed coaching schedule, cumulative coaching metrics increased with months since the start of the intervention, but coaching frequency metrics decreased over time. By design, cumulative coaching measures were positively associated with time since intervention (ρ=0.36 to 0.87) and with each other (ρ=0.30 to 0.86) while coaching frequency metrics were negatively associated with time since intervention (ρ=−0.82 to −0.97) and positively associated with each other (ρ=0.79 to 0.98) (Supplemental Table).

Fidelity at the facility level to the coaching schedule did not always translate into delivery of high-coverage coaching among birth attendants.

### EBP Adherence

In our primary model, all 4 coaching frequency metrics were significantly associated with increased EBP adherence (Table 3). On average, providing a facility with 6 coaching visits per month was associated with birth attendants adhering to an additional 1.3 EBPs (95% CI=0.6, 1.9). The association between EBP adherence and coaching frequency was larger in magnitude and if coaching was delivered with high coverage among birth attendants: providing 70% of birth attendants with at least 1 visit per month was associated with adherence to 2.0 additional EBPs (95% CI=1.0, 2.9), and providing all BAs at a facility with at least 1 visit per month was associated with adherence to 2.8 additional EBPs (95% CI=1.4, 4.2). However, no cumulative coaching or scheduling adherence metrics were significantly associated with EBP adherence. The stepwise selection procedure did not identify a model that included multiple coaching metrics.

**TABLE 3.** Association Between Coaching Intensity and EBP Adherence Among BAs During Births in 15 Health Facilities, Uttar Pradesh, India (N=2,083 Births)

Coaching Domain	Units in IQR Increase	Model 1^a^			Model 2^b^		
		Change in Practices Adhered to Associated With 1-Unit Increase (95% CI)	Change in Practices Adhered to Associated With IQR Increase (95% CI)	*P* Value	Change in Practices Adhered to Associated With 1-Unit Increase (95% CI)	Change in Practices Adhered to Associated With IQR Increase (95% CI)	*P* Value
**Coaching frequency**							
Visits in the past month	6.0	0.2 (0.1, 0.3)	1.3 (0.6, 1.9)	<.01	0.2 (−0.0, 0.4)	1.0 (−0.1, 2.2)	.10
Mean visits in the past month per BA	1.3	1.0 (0.6, 1.4)	1.2 (0.7, 1.8)	<.01	0.9 (0.2, 1.6)	1.2 (0.3, 2.1)	.01
BAs receiving ≥1 visit in past month, %	70	2.8 (1.4, 4.2)	2.0 (1.0, 2.9)	.01	3.4 (1.0, 5.8)	2.4 (0.7, 4.0)	.03
Standard deviation in visits among BAs past month	1.3	0.9 (0.5, 1.4)	1.2 (0.6, 1.8)	.01	0.7 (0.0, 1.5)	1.0 (-0.0, 1.9)	.08
**Cumulative coaching**							
Total visits	8.0	−0.0 (−0.1, 0.0)	−0.4 (−0.8, 0.1)	.09	0.1 (0.0, 0.1)	0.6 (0.3, 0.9)	.07
Mean visits per BA	5.3	−0.2 (−0.4, 0.0)	−1.0 (−2.1, 0.1)	.09	0.2 (0.0, 0.4)	1.0 (0.0, 2.0)	.21
BAs receiving ≥10 visits, %	40	−3.0 (−5.9, −0.1)	−1.2 (−2.4, 0.0)	.12	0.3 (−3.5, 4.1)	0.1 (−1.4, 1.6)	.89
Standard deviation in visits among BAs	3.5	−0.2 (−0.4, 0.1)	−0.6 (−1.5, 0.2)	.12	0.3 (0.0, 0.5)	0.9 (0.2, 1.7)	.08
**Scheduling adherence**							
Current scheduling nonadherence	NA^c^	0.3 (−0.7, 1.3)	–	.55	-0.5 (−1.3, 0.4)	–	.27
Cumulative scheduling nonadherence	12	−0.0 (−0.1, 0.0)	−0.5 (−1.0, 0.1)	.08	0.1 (0.0, 0.1)	0.8 (0.0, 1.6)	.11

Abbreviations: BA, birth attendant; CI, confidence interval; EBP, essential birth practice; IQR, interquartile range, NA, not applicable.

Effects are reported for a 1-unit increase and for increasing each continuous coaching metric from its 25^th^ percentile to its 75^th^ percentile, that is, by 1 IQR. Results are from a generalized linear model with an identity link. Standard errors are estimated using the empirical variance with an exchangeable working covariance structure to account for clustering at the facility level.

a Adjusted for whether the facility was in a high-priority district, distance to district hospital, facility staff size, facility delivery load, and whether birth occurred on the same day as a coaching visit.

b Adjusted for everything in Model 1 plus months since start of the intervention.

c Because current scheduling nonadherence is a binary outcome, we report the effect for nonadherence vs. no adherent, rather than for a 1 IQR increase.

**FIGURE 3:**
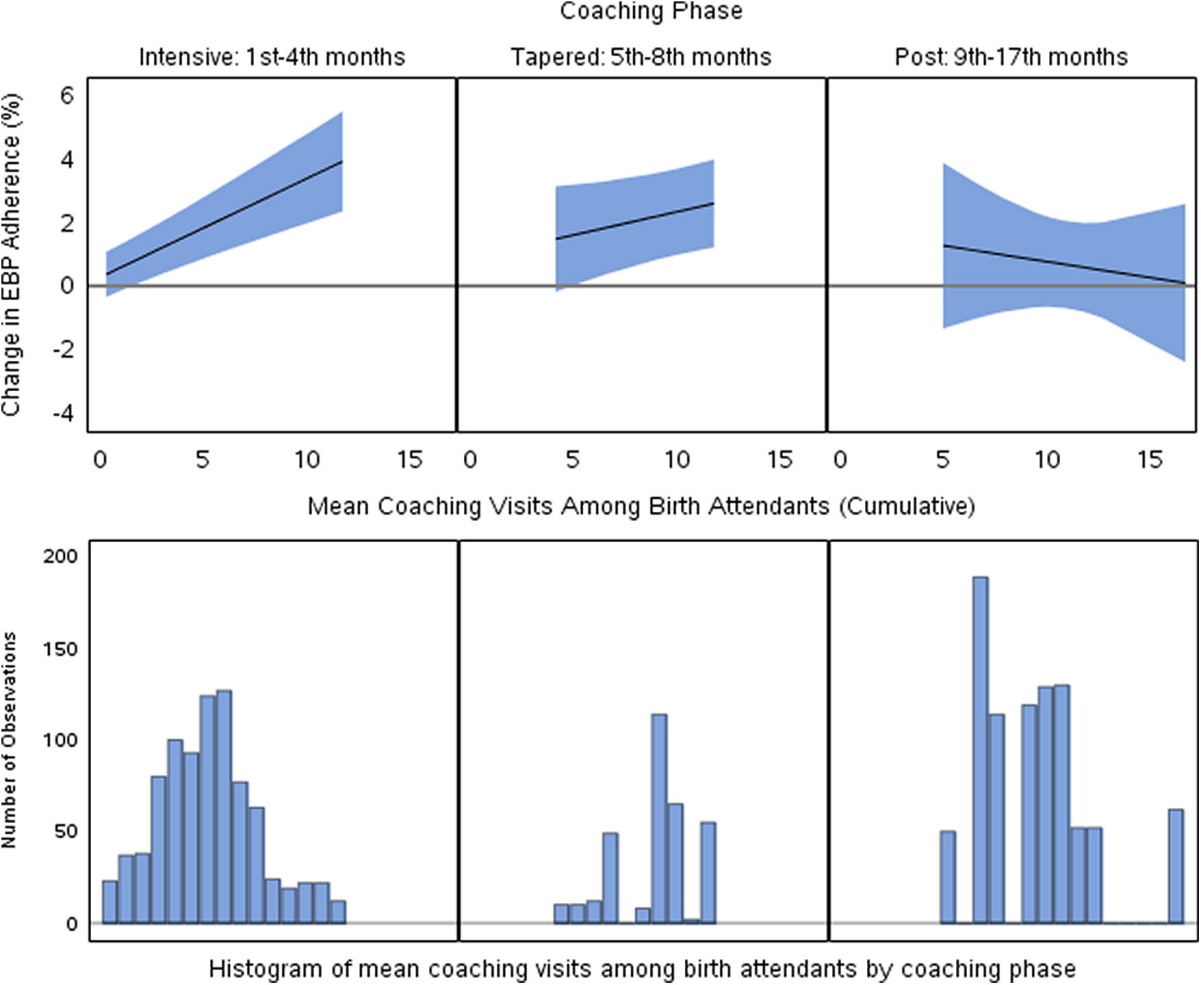
Effect Modification of the Association Between Mean Coaching Visits Among Birth Attendants (Cumulative) and EBP Adherence Over Months of the Intervention (N=2,083)^a^ Abbreviation: EBP, essential birth practice. ^a^Effect sizes for coaching phases plotted at 2, 6, 12 months since the start of the intervention. Test for interaction: *P*<.01. Overall test for all coaching terms: *P*=.04.

When we included months since the start of the intervention in our model, we did not detect any nonlinear effects of time. After adjusting for time since the start of the intervention, mean visits in the past month per birth attendant and percentage of birth attendants who received at least 1 visit in the past month, 2 coaching frequency metrics that both assessed coaching delivered at the birth attendant level, remained significantly and positively associated with increased EBP adherence. Also, cumulative coaching metrics became nonsignificantly associated with increased EBP adherence. When we included an interaction term between coaching intensity metrics and time since the start of the intervention, the effect of coaching was found to vary over time for only 1 coaching metric, mean coaching visits per birth attendant (test for interaction: *P*<.01; test for overall significance of coaching: *P*=.04; Supplemental Tables show results from additional models). This cumulative coaching measure was associated with increased EBP adherence during the early months of the intervention when coaching occurred very frequently, but the positive association did not persist after coaching visits ceased (Figure 3). There was no evidence that the association between coaching frequency metrics and EBP adherence were modified by months since the start of the intervention.

### Health Outcomes

In general, coaching was not associated with health outcomes (Table 4). In our primary model, nonadherence to the coaching schedule was associated with an increased risk of the primary composite outcome, which reflected maternal morbidity, maternal mortality, and perinatal mortality, but this result was attenuated after adjusting for months since the start of the intervention. After adjusting for months since the start of the intervention, we also observed a significant association between average visits per birth attendant and increased risk of the primary composite outcome (relative risk [RR]=1.10, 95% CI=1.03, 1.18). However, because this model also estimated an implausibly strong 18% reduction in the risk of mortality or morbidity over the course of a year (RR=0.82, 95% CI=0.71, 0.95), this association likely reflects strong correlations between time and coaching rather than a true adverse effect of coaching. Our stepwise selection procedure did not identify a model that included multiple coaching metrics, and no significant interactions were detected.

**TABLE 4.** Risk Ratios for the Association Between Coaching and Health Outcomes^a^ Among BAs During Births in Health Facilities, Uttar Pradesh, India

Coaching Domain	Units in Increase	Primary Composite Maternal Morbidity or Maternal or Infant Mortality (n/N=12,062/79,777)				Secondary Composite Maternal or Infant Mortality (n/N=3,907/80,234)			
		Model 1^b^		Model 2^c^		Model 1^b^		Model 2^c^	
		RR (95% CI)	*P* Value	RR (95% CI)	*P* Value	RR (95% CI)	*P* Value	RR (95% CI)	*P* Value
**Coaching frequency**									
Visits in past month	6.0	1.03 (0.99, 1.07)	.14	1.03 (0.94, 1.13)	.49	0.98 (0.92, 1.05)	.61	1.01 (0.88, 1.15)	.91
Mean visits in past month per BA	1.3	1.03 (1.00, 1.06)	.10	1.02 (0.97, 1.08)	.36	0.98 (0.93, 1.04)	.54	0.99 (0.92, 1.07)	.84
BAs receiving ≥1 visit in past month, %	0.7	1.05 (1.00, 1.10)	.05	1.06 (0.98, 1.15)	.15	1.00 (0.92, 1.09)	.99	1.07 (0.93, 1.22)	.36
Standard deviation in visits among BAs in past month	1.3	1.02 (0.98, 1.05)	.30	0.99 (0.94, 1.05)	.82	1.00 (0.95, 1.06)	.98	1.06 (0.99, 1.14)	.11
**Cumulative coaching**									
Total visits	8.0	0.99 (0.97, 1.02)	.50	1.02 (0.98, 1.05)	.42	1.01 (0.97, 1.05)	.59	1.00 (0.94, 1.06)	.93
Mean visits per BA	5.3	1.01 (0.96, 1.07)	.70	1.10 (1.03, 1.18)	.03	1.07 (0.98, 1.18)	.18	1.10 (0.98, 1.25)	.16
BAs receiving ≥10 visits, %	0.4	1.00 (0.96, 1.05)	.87	1.04 (0.99, 1.09)	.14	1.04 (0.96, 1.13)	.32	1.04 (0.94, 1.15)	.42
Standard deviation in visits among BAs	3.5	1.00 (0.95, 1.06)	.89	1.04 (0.98, 1.11)	.25	1.04 (0.96, 1.13)	.34	1.04 (0.96, 1.14)	.37
**Scheduling adherence**									
Current scheduling nonadherence^d^	1	1.06 (1.01, 1.12)	.04	1.05 (1.00, 1.11)	.08	0.97 (0.86, 1.09)	.58	0.98 (0.87, 1.10)	.69
Cumulative scheduling nonadherence	12	1.00 (0.96, 1.05)	.85	1.06 (0.99, 1.13)	0.10	1.03 (0.98, 1.09)	.23	1.05 (0.99, 1.12)	.12

Abbreviations: BA, birth attendant; CI, confidence interval; RR, risk ratio.

a Effects are reported for increasing each continuous coaching metric from its 25^th^ percentile to its 75^th^ percentile, that is, 1 interquartile range. Results are from a generalized linear model with a log link and binomial distribution. Standard errors are estimated using the empirical variance with an exchangeable working covariance structure.

b Adjusted for hub name, whether the facility was in a high-priority district, distance to district hospital, facility staff size, facility delivery load, whether birth occurred on the same day as a coaching visit.

c Adjusted for everything in Model 1 plus months since start of the intervention.

d Because current scheduling nonadherence is a binary outcome, we report the effect for infidelity vs. no infidelity, rather than for a 1 interquartile range increase.

## DISCUSSION

Our analysis suggests that in the BetterBirth Trial, coaching frequency was associated with modestly increased EBP adherence. Associations between coaching frequency and EBP adherence tended to be stronger when considering coaching delivered at the birth attendant level rather than the facility level. In contrast, cumulative coaching was generally not associated with EBP adherence. However, when we adjusted for time since the start of the intervention, cumulative coaching metrics became nonsignificantly positively associated with increased EBP adherence. When we allowed the effect of coaching to change over time since the start of the intervention, one cumulative coaching metric, mean visits per birth attendant, was significantly associated with increased EBP adherence during the early months of the intervention.

Associations between coaching frequency and EBP adherence were stronger when we assessed coaching delivered at the birth attendant level rather than the facility level.

Because the BetterBirth coaching schedule induced strong correlations between coaching metrics, it is difficult to isolate the independent effects of coaching frequency from cumulative coaching. Despite this limitation, our analyses suggest that high-frequency, high-coverage coaching can modestly increase birth attendant adherence to EBPs. However, the positive effects of coaching diminished over time. Since the gains in EBP adherence were not sustained after frequency tapered, future interventions seeking to promote sustained improvements in EBP adherence may consider providing high-frequency, high-coverage coaching over a longer period of time. Future researchers may also consider identifying feasible and cost-effective mechanisms for delivering this sort of high-intensity coaching as well as mechanisms for improving the sustainability of the intervention through enhanced facility-level engagement.

The main trial reported greater EBP adherence in the intervention arm (11.1 of 18.0 EBPs, 95% CI=10.4,11.8) compared to the control arm (7.9 of 18.0 EBPs, 95% CI=7.4, 8.4) 12 months into the intervention but no significant changes in health outcomes.^29^ Similarly, in the present analysis, health outcomes were generally not associated with coaching. As has been previously noted, this may reflect the fact that adherence to EBPs is an inadequate surrogate outcome for maternal and neonatal health.^50^ Alternatively, this lack of association may reflect the fact that the magnitude of the association between coaching and total EBP completion was relatively modest. This small absolute change in EBP adherence may not have produced sufficient improvements in the quality of care to impact health outcomes. We did observe an increased probability of experiencing maternal morbidity, maternal mortality, or perinatal mortality on days when sites had deviated from the intervention's prescribed coaching schedule. Because coaching was not associated with improved health outcomes in any other models, this association likely does not reflect direct benefits of coaching. Instead, it may suggest that sites that were unable to adhere to the coaching schedule were also experiencing other structural issues, such as poor leadership, understaffing, or inaccessibility, that placed mothers and infants at risk of harm.

Health outcomes were generally not associated with coaching.

Previous articles have reported dose-response relationships between coaching intensity and health worker behavior change,^21^^–^^23^ but to our knowledge, this is the first article that simultaneously investigated multiple domains of coaching intensity. Consequently, there was relatively little precedent for defining coaching intensity metrics. Although we initially expected greater variation in coaching among birth attendants to reflect poor coaching coverage and be associated with worse outcomes, greater standard deviations in visits among birth attendants in the past month was significantly associated with improved EBP adherence. This unexpected association could be explained if coaches were strategically providing additional coaching to specific birth attendants, such as the facility's designated childbirth quality coordinator. Alternatively, this metric may have been too strongly correlated with the remaining coaching frequency metrics (ρ=0.79 to 0.98) to serve as an independent metric of coaching disparities among birth attendants. Similarly, although cumulative scheduling nonadherence and cumulative standard deviation in visits among birth attendants were hypothesized to have adverse effects, both metrics were highly correlated with and produced results similar to cumulative coaching metrics hypothesized to be beneficial. In coaching-based interventions, deviations from the coaching schedule and disparities in the delivery of coaching to individual health care workers will gradually accrue over time as new opportunities for scheduling conflicts arise. Consequently, in many settings, we would expect cumulative scheduling nonadherence and standard deviation-based coaching metrics to exhibit problematic correlations with other cumulative coaching metrics that complicate their interpretation and may not be appropriate choices of coaching intensity metrics for future studies.

This is the first article that simultaneously investigated multiple domains of coaching intensity on care worker behavior change.

Our finding that frequent coaching was associated with modest improvements in EBP adherence is similar to previous reports. A recent meta-analysis found that strategies commonly used to improve health care worker performance including supervision, training, and group problem solving, are associated with improving health care worker performance by 1.0, 6.4, and 13.6 percentage points, respectively.^17^ We found that, using the BetterBirth model of coaching, providing intervention facilities with 6 coaching visits per month was associated with adherence to 1.3 additional EBPs, or a 7.2 percentage-point increase on our 18.0-point EBP adherence scale. Although the magnitude of the association between coaching and EBP adherence was relatively small, providing high-frequency, high-coverage coaching may be able to make a greater difference. Our results suggest that if all birth attendants at a facility were provided with at least 1 coaching visit per month, EBP adherence could increase by 2.8 practices, or 15.0 percentage points. Our results are similar to findings related to low-dose, high-frequency (LDHF) training, which combines brief onsite trainings with short, frequent practice sessions and has produced positive behavior change among birth attendants and improved maternal and child health outcomes.^51^^,^^52^ Although LDHF models emphasize the acquisition of new skills through training and practice rather than coaching health care providers to better use their preexisting training, both the LDHF model and our results suggest that frequent contact with health care workers may be necessary to improve quality of care and sustain the improvements.

In this study, all facilities were primary-level health facilities located in Uttar Pradesh, and all coaching was provided by external peer coaches according to the opportunity-ability-motivations-supplies framework.^33^ Consequently, we do not know the extent to which the relationships observed in this analysis can be generalized to other settings or to other forms of coaching. Contextual factors, including staffing levels, facility infrastructure, and the interactions between coaching activities and traditional supervision processes may modify the effectiveness of coaching.

Other programs that have used coaching as a Safe Childbirth Checklist implementation strategy have reported extremely variable EBP adherence at end line (range=32%–93%).^29^^,^^53^^–^^60^ In general, these interventions have not specified behavior change models nor provided full details on the frequency or duration of coaching delivered. Consequently, it is difficult to determine the extent to which observed differences in the effectiveness of these interventions result from contextual differences between study settings, differences in facilities' readiness to change, or differences in their coaching intensity. Furthermore, some of these studies relied primarily on internal coaches recruited from within intervention facilities.^53^^,^^60^ Although the intensity of external coaching interventions can be evaluated using dates of coaching visits, this approach would not apply to internal coaches who are embedded within the intervention facilities and may therefore engage in coaching for variable amounts of time each day. Alternative approaches of assessing coaching intensity, such as time-motion studies, may be more appropriate for evaluating the intensity of internal coaching strategies.^61^ Future research is needed to compare both the effectiveness and the sustainability of internal and external coaching in this setting.

Our data provide several practical insights for those seeking to implement or study future coaching-based interventions. First, even high fidelity to a facility-centric coaching schedule will not necessarily ensure that individual health care workers receive adequate coaching coverage. In our study, the unequal distribution of coaching among birth attendants likely reflect preferential behavior on the part of the coaches, dynamics related to the timing of shifts in birth facilities or are the result of staff turnover. Interventionists should specify and monitor coaching delivered at both the facility and the health care worker levels to identify whether these processes are taking place.

Second, our study suggests that high-frequency coaching can improve health worker adherence to EBPs, but maintaining high-frequency coaching is a resource-intensive intervention. Future interventionists may wish to explore cost-effective methods for maintaining high-frequency coaching over longer periods of time, such as recruiting internal coaches, coaching more consistently on health systems at a facility-level, or combining in-person coaching visits with remote coaching methods.

Although high-frequency coaching can improve health worker adherence to EBPs, it is a resource-intensive intervention.

Third, identifying optimal coaching regimens requires designing interventions that have uncorrelated variation in coaching frequency and cumulative coaching. This could be achieved, for example, by conducting a 3-armed trial with 1 control arm, 1 arm receiving evenly spaced coaching visits over a set duration of time, and a third arm receiving the same total number of coaching visits delivered over the same duration of time but following a tapered schedule similar to that of the BetterBirth Program's.

Finally, statistically significant improvements in quality of care indicators such as EBP adherence do not necessarily translate into meaningful improvements in health outcomes. In general, changes in quality of care indicators will be more likely to predict improvements in health outcomes if researchers choose quality of care indicators that are a valid surrogate for the primary health outcomes of interest, have been empirically demonstrated to have a causal relationship with the health outcome, if the magnitude of the change is clinically meaningful on the absolute scale (e.g., EBP adherence increased by 20 percentage points from 60% to 80%) rather than on the relative scale (e.g., EBP adherence doubled from 5% to 10%), and if the quality of care indicators reflect most or all major determinants of the primary health outcome.^62^ We recommend that future researchers consider whether the quality of care improvements observed in their study are large enough to plausibly generate meaningful improvements in the health outcomes. If not, some combination of more frequent coaching, additional implementation strategies, and identifying and addressing systemic barriers may be needed to further improve the quality of care and ultimately achieve the desired health impact.

### Limitations

Our analysis has several limitations. As discussed above, the BetterBirth Program's coaching schedule created strong correlations among coaching intensity metrics, which complicated the interpretation of some findings. Second, this analysis focused on 2 domains of coaching intensity, coaching frequency and cumulative coaching, but was unable to assess other domains, including coaching quality, which was unmeasured, or coaching form, which did not vary across sites. Therefore, we cannot comment on which aspects of coaching are most effective, and it is possible that alternative coaching models, such as those that coach on clinical quality rather than on adherence to certain tasks, may be more effective than the model used here. We also did not capture information on the duration or timing of individual coaching visits.

Third, although we sought to minimize bias by adjusting for facility-level characteristics, residual confounding is possible if unmeasured facility or birth attendant characteristics that were associated with the outcomes also impacted coaches' behavior. For example, reports suggest that, to minimize travel time, coaches would provide difficult-to-reach facilities with visits on back-to-back days. If less accessible facilities experienced worse outcomes, we would expect this practice to bias our results against coaching frequency metrics. Coaches may have also been more likely to provide coaching to the facilities or birth attendants who were most motivated and receptive to their help and less likely to provide coaching to relatively junior nurses, who often staff evening, night, and weekend shifts. We would expect both of these processes to bias our results in favor of coaching.

Fourth, assessment of EBP adherence outcomes was based on directly-observed sessions that took place during daylight hours and may not reflect adherence at night or when birth attendants were not observed. Finally, because we were unable to link individual birth attendants to individual births in our dataset, we assessed the effectiveness of coaching delivered at the birth attendant level using facility-level aggregated metrics that did not consider staff turnover within the health facility. We would expect this measurement error to result in an underestimation of the direct benefits of providing coaching to individual birth attendants.

## CONCLUSIONS

Frequent coaching was associated with increased adherence to essential birth practices among birth attendants in the BetterBirth Trial. The effect size was greater for coaching delivered at the birth attendant level compared to coaching delivered at the facility level. Cumulative coaching metrics were not associated with essential birth practice adherence, suggesting that the short-term effects of high-frequency coaching may not translate into sustained effects of cumulative coaching over time. Future coaching-based interventions seeking to promote sustainable change may need to consider identifying sustainable, cost-effective models for providing more frequent, high-coverage coaching for longer periods. Coaching was generally not associated with health outcomes, suggesting that additional coaching and other implementation strategies may be needed to achieve the desired health impact.

## Supplementary Material

19-00317-Barnhart-Supplement_Tables.pdf

## Notes

**Peer Reviewed**

**First published online:** March 3, 2020

**Availability of data and material:** Data from this study are available at the Harvard Dataverse repository at Barnhart DA, Spiegelman D, Zigler CM, et al. Coaching intensity, adherence to essential birth practices, and health outcomes in the BetterBirth Trial. Harvard Dataverse, V2. https://doi.org/10.7910/DVN/ONRYVC.

**Funding:** This study was funded by the U.S. National Institute of Environmental Health Sciences (grant #5DP1ES025459) and the Bill & Melinda Gates Foundation (grant #OPP1017378). The funders were not involved with study design; data collection, analysis, and interpretation; report writing; or in the decision to submit findings for publication.
