# Supplementary material for: Coaching Intensity, Adherence to Essential Birth Practices, and Health Outcomes in the BetterBirth Trial in Uttar Pradesh, India
Source: Glob Health Sci Pract. 2020 Mar 30;8(1):38–54. doi: 10.9745/GHSP-D-19-00317 (PMC7108945; doi:10.9745/GHSP-D-19-00317)
Supplement: 19-00317-Barnhart-Supplement_Tables.pdf [file 19-00317-Barnhart-Supplement_Tables.pdf]

Supplement to: Barnhart DA, Spiegelman D, Zigler CM, et al. Coaching intensity, adherence to essential birth practices, and health outcomes in the BetterBirth Trial. *Glob Health Sci Pract*. 2020;8(1). https://doi.org/10.9745/GHSP-D-19-00317

Supplemental Table 1. Spearman Correlation Coefficients Between Coaching Metrics and Time Since Start of the Intervention

In each cell, the top number reflects the correlation coefficient in the birth attendant (BA) essential birth practice (EBP) adherence dataset (N=2,083) and the bottom number reflects the correlation coefficient in the health outcomes dataset (N=80,234). Bolding is used to highlight groups of cumulative coaching and coaching frequency variables.

	**A**	**B**	**C**	**D**	**E**	**F**	**G**	**H**	**I**	**J**	**K**	**L**	**M**	**N**	**O**
Months since start of intervention (A)	1.00	-.85	-.87	-.85	-.87	-.82	-.81	-.81	-.81	0.81	0.65	0.57	0.60	-.23	0.77
	1.00	-.97	-.93	-.82	-.92	-.83	-.79	-.77	-.80	0.87	0.50	0.42	0.36	-.23	0.71
Visits in past month (B)		1.00	**0.96**	**0.92**	**0.96**	**0.90**	**0.88**	**0.86**	**0.88**	-.78	-.55	-.50	-.43	0.16	-.71
		1.00	**0.96**	**0.85**	**0.95**	**0.86**	**0.82**	**0.80**	**0.83**	-.85	-.47	-.38	-.33	0.19	-.71
Mean visits in past month per BA (C)			1.00	**0.95**	**0.98**	**0.89**	**0.91**	**0.89**	**0.90**	-.80	-.48	-.45	-.37	0.17	-.70
			1.00	**0.93**	**0.94**	**0.82**	**0.83**	**0.81**	**0.82**	-.80	-.32	-.25	-.28	0.19	-.67
BAs receiving ≥1 visit in past month, % (D)				1.00	**0.89**	**0.86**	**0.86**	**0.87**	**0.84**	-.78	-.50	-.50	-.49	0.18	-.68
				1.00	**0.79**	**0.71**	**0.73**	**0.73**	**0.71**	-.69	-.23	-.16	-.30	0.17	-.57
Standard deviation in visits among BAs in past month (E)					1.00	**0.87**	**0.88**	**0.85**	**0.89**	-.79	-.51	-.46	-.35	0.19	-.69
					1.00	**0.81**	**0.79**	**0.77**	**0.81**	-.80	-.37	-.30	-.21	0.18	-.65
Visits in past week (F)						1.00	**0.97**	**0.95**	**0.97**	-.79	-.56	-.56	-.44	-.05	-.74
						1.00	**0.97**	**0.94**	**0.97**	-.76	-.44	-.36	-.32	-.08	-.66
Mean visits in past week per BA (G)							1.00	**0.98**	**0.98**	-.78	-.49	-.49	-.37	-.04	-.72
							1.00	**0.99**	**0.98**	-.71	-.34	-.27	-.27	-.07	-.61
As receiving ≥1 visit in past week, % (H)								1.00	**0.95**	-.77	-.50	-.50	-.41	-.02	-.71
								1.00	**0.95**	-.69	-.31	-.25	-.26	-.06	-.59
Standard deviation in visits among BAs in past week (I)									1.00	-.77	-.50	-.49	-.36	-.04	-.71
									1.00	-.72	-.36	-.29	-.27	-.07	-.62
Total visits (J)										1.00	**0.64**	**0.60**	**0.53**	-.22	0.60
										1.00	**0.48**	**0.38**	**0.38**	-.22	0.62
Mean visits per BA (K)											1.00	**0.83**	**0.78**	-.19	0.55
											1.00	**0.86**	**0.52**	-.12	0.44
BAs receiving ≥10 visits, % (L)												1.00	**0.61**	-.17	0.57
												1.00	**0.30**	-.10	0.35
Standard deviation in visits among BAs (M)													1.00	-.18	0.46
													1.00	-.08	0.35
Current scheduling nonadherence (N)														1.00	-.08
														1.00	-.13
Cumulative scheduling nonadherence (O)															1.00
															1.00

Supplement to: Barnhart DA, Spiegelman D, Zigler CM, et al. Coaching intensity, adherence to essential birth practices, and health outcomes in the BetterBirth Trial. *Glob Health Sci Pract*. 2020;8(1). https://doi.org/10.9745/GHSP-D-19-00317

Supplemental Table 2. Coefficients for Models Testing the Effect Modification of Coaching Intensity by Months Since Start of the Interaction

	**EBP Adherence** N=2,083 births			**Primary Composite** Maternal morbidity, maternal or infant mortality (n/N=12,062/79,777)			**Secondary Composite** Maternal or infant mortality (n/N=3,907/80,234)		
**Coaching Intensity**	**Main effect of coaching**	**Main effect of months since start of intervention**	**Interaction Term**	**Main effect of coaching**	**Main effect of months since start of intervention**	**Interaction Term**	**Main effect of coaching**	**Main effect of months since start of intervention**	**Interaction Term**
**Coaching frequency (past month)**									
Visits in the past month	-0.04	-0.03	0.08	-0.001	0.004	0.003	-0.01	0.01	0.005
Mean visits in the past month per BA	-0.02	-0.01	0.34	-0.01	0.001	0.01	-0.06	0.004	0.02
BAs receiving ≥1 visit in past month, %	1.52	0.01	0.52	0.08	0.003	0.001	-0.08	0.003	0.02
Standard deviation in visits among BAs in past month	-0.15	-0.03	0.33	-0.02	-0.01	0.01	0.02	0.01	0.01
**Coaching frequency (past week)**									
Visits past week	-0.35	-0.10	0.22	-0.05	-0.01	0.01	-0.07	0.003	0.02
Mean visits in past week per BA	-0.60	-0.08	0.82	-0.14	-0.01	0.04	-0.27	-0.0001	0.07
BAs receiving ≥1 visit in past week, %	-0.38	-0.09	0.91	-0.14	-0.01	0.04	-0.22	0.003	0.07
Standard deviation in visits among BAs in past week	-0.91	-0.11	0.55	-0.12	-0.01	0.03	-0.15	0.004	0.05
**Cumulative coaching**									
Total visits	0.08	0.92	-0.03	0.01	0.19	-0.004	0.004	0.13	-0.003
Mean visits per BA	0.39	0.15	-0.04	0.02	-0.01	-0.0001	0.01	-0.02	0.001
BAs receiving ≥10 visits, %	3.79	0.09	-0.64	0.10	0.009	-0.001	0.06	-0.004	0.01
Standard deviation in visits among BAs	0.42	-0.07	-0.02	0.01	-0.01	0.0000	0.01	-0.001	0.0001
**Scheduling adherence**									
Current scheduling nonadherence	-3.05	-0.15	0.70	0.06	-0.004	-0.002	0.12	0.005	-0.03
Cumulative scheduling nonadherence	0.05	-0.35	0.003	0.001	-0.04	0.001	0.004	-0.01	0.0002

Abbreviations: BA, birth attendant; EBP, essential birth practice.

Supplement to: Barnhart DA, Spiegelman D, Zigler CM, et al. Coaching intensity, adherence to essential birth practices, and health outcomes in the BetterBirth Trial. *Glob Health Sci Pract*. 2020;8(1). https://doi.org/10.9745/GHSP-D-19-00317

Supplemental Table 3. Effect Modification of Coaching Intensity by Months Since Start of the Interaction

The *P* value of the overall significance of coaching reflects the joint null hypothesis that both the main effect of coaching and the coaching-by-time interaction term are equal to 0.

	**EBP Adherence** N=2,083 births		**Primary Composite** Maternal Morbidity, Maternal or Infant Mortality (n/N=12,062/79,777)		**Secondary Composite** Maternal or Infant Mortality (n/N=3,907/80,234)	
	**Interaction Term**	**Overall Significance of Coaching**	**Interaction Term**	**Overall Significance of Coaching**	**Interaction Term**	**Overall Significance of Coaching**
**Coaching Intensity**	***P* Value**	***P* Value**	***P* Value**	***P* Value**	***P* Value**	***P* Value**
**Coaching frequency (past month)**						
Visits in the past month	.09	.62	.09	.82	.11	.71
Mean visits in the past month per BA	.08	.28	.09	.94	.11	.25
BAs receiving ≥1 visit in past month, %	.06	.15	.97	.38	.26	.70
Standard deviation in visits among BAs in past month	.07	.53	.32	.48	.44	.49
**Coaching frequency (past week)**						
Visits past week	.07	.43	.02	.06	.02	.14
Mean visits in past week per BA	.11	.74	.01	.10	.04	.08
BAs receiving ≥1 visit in past week, %	.09	.64	.04	.28	.05	.34
Standard deviation in visits among BAs in past week	.09	.52	.02	.15	.03	.31
**Cumulative coaching**						
Total visits	.23	.09	.12	.22	.09	.77
Mean visits per BA	<.01	.04	.91	.03	.54	.43
BAs receiving ≥10 visits,%	.02	.11	.98	.26	.79	.68
Standard deviation in visits among BAs	.05	.04	.97	.31	.98	.45
**Scheduling adherence**						
Current scheduling nonadherence	.04	.06	.92	.46	.44	.54
Cumulative scheduling nonadherence	.63	.14	.07	.53	.85	.39

Abbreviations: BA, birth attendant; EBP, essential birth practice.

Supplement to: Barnhart DA, Spiegelman D, Zigler CM, et al. Coaching intensity, adherence to essential birth practices, and health outcomes in the BetterBirth Trial. *Glob Health Sci Pract*. 2020;8(1). https://doi.org/10.9745/GHSP-D-19-00317

Supplemental Table 4. Effects of Coaching Frequency Calculated Using a 1-Week Time Horizon on EBP Adherence

Effects are reported for increasing each continuous coaching metric from its 25th percentile to its 75th percentile, or by 1 interquartile range (IQR). Results are from a generalized linear with an identity link. Standard errors are estimated using the empirical variance with an exchangeable working covariance structure to account for clustering at the facility level. (N=2,083 births)

		Model 1[a]			Model 2[b]		
**Coaching Intensity**	**Units in IQR increase**	**Δ in practices adhered to associated with 1-unit increase (95% CI)**	**Δ in practices adhered to associated with IQR increase (95% CI)**	***P* Value**	**Δ in practices adhered to associated with 1-unit increase (95% CI)**	**Δ in practices adhered to associated with IQR increase (95% CI)**	***P* Value**
Coaching frequency							
Visits past week	1.0	0.7 (0.3, 1.0)	0.7 (0.3, 1.0)	<.01	0.3 (-0.1, 0.6)	0.3 (-0.1, 0.6)	.14
Mean visits in past week per BA	0.3	2.8 (1.5, 4.2)	0.8 (0.4, 1.2)	.01	1.6 (0.2, 3.0)	0.5 (0.1, 0.9)	.03
BAs receiving ≥1 visit in past week, %	30	4.0 (2.0, 6.0)	1.2 (0.6, 1.8)	.01	2.2 (0.3, 4.1)	0.7 (0.1, 1.2)	.04
Standard deviation in visits among BAs in past week	0.5	2.0 (0.9, 3.0)	1.0 (0.5, 1.5)	.01	0.8 (-0.3, 1.9)	0.4 (-0.2, 0.9)	.14

Abbreviations: BA, birth attendant; CI, confidence interval, IQR, interquartile range.

[a] Adjusted for whether the facility was in a high-priority district, distance to district hospital, facility staff size, facility delivery load, whether birth occurred on the same day as a coaching visit.

[b] Adjusted for everything in Model 1 plus months since start of the intervention (no non-linear effects of time detected).

Supplement to: Barnhart DA, Spiegelman D, Zigler CM, et al. Coaching intensity, adherence to essential birth practices, and health outcomes in the BetterBirth Trial. *Glob Health Sci Pract*. 2020;8(1). https://doi.org/10.9745/GHSP-D-19-00317

Supplemental Table 5. Effects of Coaching Frequency Calculated Using a 1-Week Time Horizon on Health Outcomes

Effects are reported for increasing each continuous coaching metric from its 25th percentile to its 75th percentile, or by 1 interquartile range (IQR). Results are from a generalized linear model with a log link and binomial distribution. Standard errors are estimated using the empirical variance with an exchangeable working covariance structure.

		**Primary Composite** Maternal morbidity or maternal or infant mortality (n/N=12,062/79,777)				**Secondary Composite** Maternal or infant mortality (n/N=3,907/80,234)			
		**Model 1[a]**		**Model 2[b]**		**Model 1[a]**		**Model 2[b]**	
**Coaching Intensity**	**Units in 1 IQR**	**RR (95% CI)**	***P* Value**	**RR (95% CI)**	***P* Value**	**RR (95% CI)**	***P* Value**	**RR (95% CI)**	***P* Value**
**Coaching frequency**									
Visits past week	1.0	1.01 (0.99, 1.03)	0.41	0.99 (0.97, 1.02)	.71	0.99 (0.95, 1.03)	.69	1.00 (0.95, 1.06)	.93
Mean visits in past week per BA	0.3	1.01 (0.99, 1.04)	0.27	1.00 (0.98, 1.03)	.82	0.99 (0.95, 1.03)	.70	1.00 (0.96, 1.05)	.97
BAs receiving ≥1 visit in past week, %	0.3	1.02 (0.99, 1.06)	0.18	1.01 (0.98, 1.05)	.40	1.01 (0.95, 1.07)	.76	1.03 (0.97, 1.10)	.30
Standard deviation in visits among BAs in past week	0.5	1.02 (0.99, 1.05)	0.21	1.01 (0.97, 1.05)	.65	1.00 (0.95, 1.05)	.99	1.03 (0.96, 1.10)	.40

Abbreviations: BA, birth attendant; CI, confidence interval, IQR, interquartile range.

[a] Adjusted for whether the facility was in a high-priority district, distance to district hospital, facility staff size, facility delivery load, whether birth occurred on the same day as a coaching visit.

[b] Adjusted for everything in Model 1 plus months since start of the intervention (no nonlinear effects of time detected).
